# Primary hyperoxaluria type 3: from infancy to adulthood in a genetically unique cohort

**DOI:** 10.1007/s00467-024-06536-w

**Published:** 2024-10-30

**Authors:** Michal Julius, Hadas Shasha Lavsky, Limor Kalfon, Nehama Cohen Kfir, Miriam Herskovits, Irith Wiesmann, Tzipora C. Falik Zaccai

**Affiliations:** 1 Azrieli Faculty of Medicine, Bar Ilan University, Safed, Israel; 2 Pediatric Nephrology Unit, Galilee Medical Center, Nahariya, Israel; 3 Galilee Medical Center, Institute of Human Genetics, Nahariya, Israel; 4 Department of Radiology, Galilee Medical Center, Nahariya, Israel

**Keywords:** Primary hyperoxaluria type 3 (PH3), *HOGA1*, Oxalate, Kidney failure, Kidney stone disease

## Abstract

**Background:**

Primary hyperoxaluria type 3 (PH3) is a rare autosomal recessive disorder caused by bi-allelic genetic variants in the 4 hydroxy-2 oxoglutarate aldolase (*HOGA-1*) gene. We report the natural history of PH3 in a 16-patient cohort, 15 from a unique genetically isolated population.

**Methods:**

This retrospective single-center study followed PH3 patients between 2003 and 2023 with demographic, clinical, radiographic, genetic, and biochemical parameters. Genetic population screening was performed in four villages to determine carrier frequency and identify couples at risk in a genetically isolated population.

**Results:**

Sixteen patients with biallelic (or homozygous) pathogenic variants (PV) in *HOGA-1* (c.944_946 del, c.119C > A, c.208C > T) were included in the study, 15 Druze and one Jewish, aged 0–63 years at diagnosis (4 adults and 12 pediatric patients). All symptomatic patients had clinical or imaging signs of nephrolithiasis. One developed chronic kidney disease (CKD) stage 5; biopsy showed focal mesangial sclerosis and chronic tubulo-interstitial changes with few oxalate deposits. Two other patients had CKD stage 2 (eGFR 87 and 74 mL/min/1.73 m^2^) upon their last visit. The remaining cohort showed preserved kidney function until the latest follow-up. Of 1167 healthy individuals screened, 90 carriers were found, a rate of 1:13 in the genetically unique cohort screened.

**Conclusions:**

A high prevalence of PH3 patients was found among a unique cohort, but probably still underdiagnosed due to relatively mild disease course. The carrier rate is high. There is no specific therapy for PH3, but early diagnosis can prevent redundant diagnostic efforts and provide early treatment for kidney stone disease. Even in our homogeneous cohort, kidney stone disease severity and CKD degree were variable, supporting a suspected contribution of yet unknown genetic or environmental factors.

**Graphical abstract:**

**Figure:**
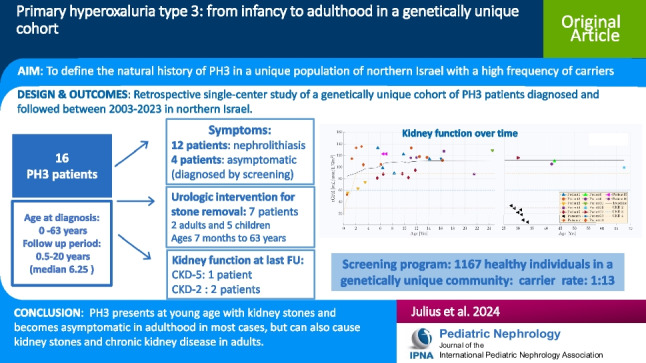
A higher resolution version of the Graphical abstract is available as Supplementary information

**Supplementary Information:**

The online version contains supplementary material available at 10.1007/s00467-024-06536-w.

## Introduction

Primary hyperoxaluria type 3 (PH3) is a rare autosomal recessive (AR) disease (OMIM#613616) that manifests as urolithiasis and nephrocalcinosis with calcium oxalate stones usually in early childhood, before the age of 5 years. PH3 usually does not lead to advanced chronic kidney disease (CKD) and systemic oxalosis [1]. Increased urine oxalate excretion and a significant family history (a sibling or cousin diagnosed with PH3) support the diagnosis that is confirmed by the determination of a bi-allelic pathogenic genetic variant (PV) in the gene 4-hydroxy-2-oxoglutarate aldolase (*HOGA*1) [2]. The long-term clinical manifestations of PH3 are unclear. The lack of data regarding the natural history of the disease is due to the novelty of its discovery only 14 years ago [2]. The largest cohorts of PH3 reported to date are from the OxalEurope consortium that included 95 PH3 patients and the rare kidney stone consortium that included 62 PH3 patients. In the OxalEurope cohort, urinary oxalate excretion was comparable to primary hyperoxaluria type 1 (PH1) and primary hyperoxaluria type 2 (PH2). CKD stage 2 or higher was found in 21.4% of the patients [3, 4]. The authors concluded that PH3 has a more favorable outcome than PH1 and PH2 but is not a benign disease as it presents in early life and can lead to CKD in adulthood. In the rare kidney stone consortium cohort, 97% of the PH3 patients did not reach CKD stage 5 until the age of 40 years, compared to 36% of PH1 and 66% of PH2 patients. Other case reports found that CKD stage 5 may develop later in life with lifelong stone events, including a case report documenting a 78-year-old man retrospectively diagnosed with PH3 after a 30-year history of urolithiasis, who went on to develop kidney failure after a nephrectomy due to clear cell papillary kidney carcinoma [5–7].

The purpose of the current study was to define the natural history of PH3 in a unique population in northern Israel with a high frequency of carriers and patients carrying a founder genetic variant.

## Methods

### Patients and families

**Fig. 1:**
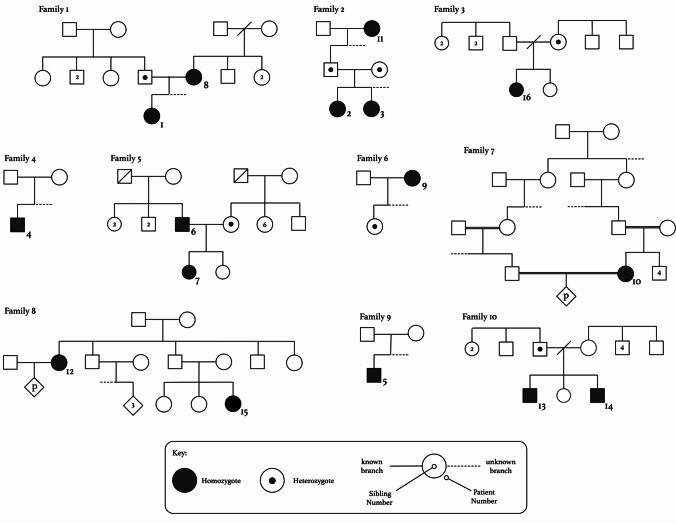
The pedigrees of the 10 families reported in this study

This retrospective, observational, single-center study includes all PH3 patients who were diagnosed and followed in the pediatric nephrology unit and the genetic institute of the Galilee Medical Center, in the north of Israel, between 2003 and 2023 (Fig. 1).

Data from the patients’ medical files were evaluated and included demographic characteristics, clinical features, and the results of genetic and other laboratory evaluation and imaging. The follow-up period was the time from the first symptoms or first diagnosis (the earlier) until December 2023 or the last follow-up visit.

### Laboratory evaluation

Regular laboratory follow-up included complete blood count, biochemistry (creatinine, electrolytes), and urinalysis. Serum creatinine has been analyzed by the Jaffe or enzymatic method in the pediatric population since 2012 and via the Jaffe method prior to 2012 [8]. We used the Abbott kit for Alinity reagent for both methods, standardized to IDMS. The eGFR was calculated using the 2009 bedside Schwartz formula (eGFR mL/min/1.73 m^2^) for patients under 18 years old, and the CKD–EPI formula for adult patients [9, 10].

### Urine oxalate testing

Urine samples for oxalate, calcium, and creatinine were evaluated from a 24-h collection or random urine sample and were interpreted according to the patient’s age [11].

Urine oxalate levels were measured in different laboratories that used different methods, either GCMS analysis or Trinity Biotech assay (enzymatic method). When possible, urine collection for oxalate was done (normal range: < 0.5 mmol or 45 mg/1.73 m^2^/day) [12]. Spot urine samples were analyzed for urinary oxalate-to-creatinine ratio (Uox/cre) in infants and young children (normal range is age-dependent: 0–0.5 year: < 0.175 mmol/mmol; 0.5–1 year: < 0.139 mmol/mmol; 1–2 years: < 0.103 mmol/mmol; 2–3 years: < 0.08 mmol/mmol; 3–5 years: < 0.064 mmol/mmol; 5–7 years: < 0.056 mmol/mmol; 7–17 years: < 0.048 mmol/mmol according to Mayo clinic laboratories reference values [13, 14].

Urine metabolite levels were either not determined (HOG, DHG, or 4OHGlu) or were not available in the patients’ medical files.

### Genetic analysis

Patients were studied by enzyme restriction analysis for the founder PV c.944_946del (RCV000000046) and the PV C.208C > T (RCV000186476) in *HOGA1*. In symptomatic heterozygotes, we performed whole genome sequencing using library construction with Truseq DNA, and library prep kit on the NovaSeq6000 platform (DNA Link, South Korea) to determine or exclude compound heterozygosity for *HOGA1* and the possible diagnosis of PH2.

We also performed Sanger sequencing of the *AGXT* coding region (NM_000030.3) to rule out PH1 in all patients. Patients found to be homozygous or compound heterozygotes for *AGXT* PV or who had other known kidney stone disease were excluded from the study. All our patients were genetically diagnosed with either PH1 or PH3, so there was no doubt about the diagnosis.

We did not exclude PH2 by genetic studies in our cohort. PH2 is ultra-rare in Israel; to the best of our knowledge, only one Israeli patient has been found to have PH2 (unpublished data, personal communications from within the Israeli Society of Pediatric Nephrology).

### Genetic screening

Population genetic screening was performed in healthy women from four villages of origin, under the regulations of the Israeli Ministry of Health (clinical public services). We approached healthy women of childbearing age in prenatal clinics from 2013 to 2022 in our search for as-yet-unidentified carriers. After counseling and their consent to testing, blood was drawn, and DNA was extracted. Population screening for c.944_946del and C.208C > T in *HOGA1* was performed by amplified fragment length polymorphism (AFLP) method using forward primer labelled with FAM. The amplified fragments were separated and visualized on an ABI 3500 fragment analyzer.

## Results

### Study cohort

Between 2003 and 2023, 16 PH3 patients (Fig. 1) were genetically diagnosed and followed up in the nephro-genetic clinic. Five patients were diagnosed in adulthood (age 23–56 years) and the remaining 11 in early childhood; 15 patients were Druze, and one was Ashkenazi Jew. Follow-up period ranged from 0.5 to 20 years, with a median of 6.25 years. One patient (patient 6) was diagnosed at the age of 29 years but had had symptoms of nephrolithiasis since the age of 1 year, so he was followed for 33 years, but this was an exceptional case (Table 1).

**Table 1:** Demographic and clinical characteristics of the reported cohort of PH3

Parameter	*N* = 16
Female gender	11 (68.7%)
Ethnicity—Druze, *N* (%)	15 (93.7%)
Age at genetic diagnosis, years, median (range)	11.55 (0–63)
Age at onset of symptoms, years, median (range)	1 (0.16–56)
First symptom:	
Asymptomatic, *N* (%)	4 (25%)
Kidney stones	11 (68.8%)
Urinary tract infection	2 (12.5%)
Nephrocalcinosis (during follow-up)	1 (6.25%)
eGFR at last follow-up, mL/min/1.73 m^2^, median (range)	109.05 (74.1–123)
Family history of PH3, *n* (%)	9 (56.2%)

### Clinical manifestations

Demographic and clinical characteristics of the reported cohort of PH3 patients are presented in Table 1 and include asymptomatic patients, and patients with recurrent nephrolithiasis at early age and until adulthood. Table 2 includes the demographic, genetic, clinical, and presenting symptoms of individuals within the cohort, while Table 3 presents the biochemical and radiological outcomes of individuals within the cohort including the clinical outcome and age at last stone events.

**Table 2:** Demographic, genetic, clinical, and presenting symptoms of individuals within the cohort

Family	Patient number	Year of birth	Gender	Ethnicity background	Age of onset of symptoms (years)	Age at genetic diagnosis (years)	Follow-up (years)	Genetic variant*	Initial symptoms
1	1	2008	Female	Druze	2	6.8	12.9	c.944_946delAGG	Nephrolithiasis, UTI
2	2	2004	Female	Druze	1	9.4	15.3	c.944_946delAGG	Urolithiasis
2	3	2014	Female	Druze	0.5	0.6	5.5	c.944_946delAGG	Nephrolithiasis Nephrocalcinosis
4	4	2002	Male	Druze	0.6	0.6	21	c.944_946delAGG/ c.119C > A ( p.Pro40His)	Urolithiasis and Nephrolithiasis
9	5	2006	Male	Jewish	1	4.2	13.3	c.944_946delAGG	Hematuria, Nephrolithiasis
5	6	1984	Male	Druze	1	29.3	33	c.944_946delAGG	Urolithiasis, UTI
5	7	2015	Female	Druze	0.5	Amniocentesis	6.3	c.944_946delAGG	Nephrolithiasis
1	8	1977	Female	Druze	-	43	NA	c.944_946delAGG	None
6	9	1978	Female	Druze	37	31.6	3	c.944_946delAGG	Prenatal tests
7	10	1994	Female	Druze	23	23	1	c.944_946delAGG	Prenatal tests
2	11	1951	Female	Druze	56	63	11	c.944_946delAGG	Obstructive bilateral Ureterolithiasis
8	12	1988	Female	Druze	29	29.7	0.5	c.944_946delAGG	Prenatal tests
12	13	2006	Male	Druze	0.8	13.3	15.8	c.208C > T p.(Arg70*)	Urolithiasis
12	14	2019	Male	Druze	0.2	3.2	3	c.208C > T p.(Arg70*)	Urolithiasis
11	15	2015	Female	Druze	2.5	4.9	4.8	c.944_946delAGG	Urolithiasis
13	16	2010	Female	Druze	1	9.8	11	c.944_946delAGG	Urolithiasis

^*^All the patients were found to be homozygous except one compound heterozygote

**Table 3:** Clinical, biochemical, and radiological outcomes of individuals within the cohort. See references values for urinary oxalate under the methods section - laboratory evaluation below

		Biomarkers [presentation / last value]			At last visit				Notes			
Family	Patient number	Uox 24-h collection mg/24 h	Uox urine sample mmol/L	Urinary Ox/cre mmol/mmol	Age (years)	Serum creatinine (mg/dl)	Height (cm)	eGFR (mL/min/1.73 m^2^)	Procedures (Nº) during follow-up	Age (years) at last stone event (defined as clinical symptoms and sonographic findings of kidney stone)	US findings at last visit	Clinical outcomes
1	1	56/45.4	310/74	-/0.02	14.9	0.63	160	114.3	None	6.3	Bilateral nephrolithiasis	Recurrent urolithiasis, medicated on potassium citrate and nitrofurantoin
2	2	-/-	145.3-	-/-	16.3	0.66	165	112.5	Ureteroscopy, ESWL (2)	4.3	Normal	Asymptomatic
2	3	-/-	-/-	-/0.71	6.1	0.54	97	74.2	None	Never	Unilateral nephrolithiasis	Nephrolithiasis on US Nephrocalcinosis – resolved
4	4	-/65	-/19.1	-/0.009	21.5	0.61 (16.5 y) 1.19 (21.5 y)	171	88	Ureteroscopy	20	Unilateral Nephrolithiasis	Recurrent urolithiasis, CKD 2
9	5	85/38.2	-/460	-/-	14.3	0.69	159	95.2	ESWL (2)	2.3	Unilateral nephrolithiasis	Urolithiasis on US
5	6	-/-	-/-	-/-	34.3	7.7	180	6.3	Double J uretric stenting	4	Unilateral nephrolithiasis	Focal mesangial sclerosis, CKD 5, dialysis, and kidney transplant
5	7	-/-	-/-	0.243/-	6.8	0.43	110	105.6	None	No symptoms of renal colic Recurrent UTI	Bilateral nephrolithiasis	Recurrent urolithiasis on US and UTI
1	8	-/-	-/-	-/-	43	0.64	-	110	None	Never	_-	Asymptomatic
6	9	68/-	-/-	-/-	42	0.71	157	105	None	Never	Normal	Asymptomatic
7	10	-/-	-/-	-/-	24.5	0.58	-	129	None	Never	_-	Asymptomatic
2	11	-/-	-/-	-/-	63	0.62	156	93.3	Bilateral ESWL	63	Bilateral nephrolithiasis	Recurrent urolithiasis
8	12	-/-	-/-	-/-	_30.1	0.55	-	125.9	None	Never	Normal	Asymptomatic
12	13	1.2/- mmol/24 h	-/-	-/-	16.5	0.84	177	115.9	None	1	Unilateral nephrolithiasis	Asymptomatic
12	14	-/-	-/-	-/0.088	3.2	0.41	95	104.3	Meatotomy for stone removal	2.7	Unilateral nephrolithiasis	Recurrent urolithiasis
11	15	-/-	-/-	-/0.183	7.3	0.47	128.5	123	Ureteroscopy for stone removal	6.9	Bilateral nephrolithiasis	Recurrent nephrolithiasis and urological interventions
13	16	0.4/- mmol / 32.4 mg	-/-	-/-	12	0.59	153	116.7	None	3	Unilateral nephrolithiasis	Asymptomatic

The median age of onset of symptoms was 1 year (range 2 months–56 years). All symptomatic patients except one were diagnosed in infancy. One patient was diagnosed following a kidney stone event at the age of 56 years. Eleven children and one adult presented with urolithiasis (Table 1).

Four asymptomatic females were diagnosed through genetic population screening in their village and continued to deny symptoms until the last follow-up (patients 8, 9, 10, 12). One affected child was diagnosed prenatally in amniocentesis upon request of her parents who were known PH3 carriers. She presented with kidney stones at age 6 months (patient 7, Table 2).

One patient (patient 6, Table 2) had CKD stage 5 and recently underwent kidney transplantation. Kidney biopsy revealed focal mesangial sclerosis and focal chronic tubulo-interstitial changes.

The oldest homozygous patient in the cohort presented with recurrent nephrolithiasis and urolithiasis from age 56 and onward through 7 years of follow-up.

### Laboratory findings

Nine patients (out of 11) were documented with high levels of urinary oxalate. We are lacking the results of urinary oxalate for the remaining five patients (see Table 3). Urinary calcium excretion from 24-h urine collections or spot urinary calcium-to-creatinine ratio was consistently within the normal range.

### Sonographic findings

Ultrasound studies were performed in our imaging department for most patients at least once a year and in most cases more frequently, depending on symptoms. We reviewed the imaging that was done in our institution to confirm the diagnosis of nephrocalcinosis or nephrolithiasis.

All symptomatic patients had some degree of nephrolithiasis/urolithiasis at some point throughout follow-up, despite not always reporting symptoms.

In two-thirds of patients, there was a tendency for the same kidney to be affected (Table 2).

Three affected children (patients 2, 3, 5; Table 2) had kidney stones on presentation and improved during follow-up until clearance of the stones and normal kidneys on ultrasound on the last follow-up (at the age of 6–14 years). One patient (#3) had mild medullary nephrocalcinosis on presentation which resolved on the last follow-up. Patients 8, 9, 10, and 12 who were diagnosed through screening and denied symptoms did not undergo imaging studies.

### Urologic intervention

Seven patients (patients 2, 4, 5, 6, 11, 14, 15) required urologic intervention — ureteric stent or extracorporeal shockwave lithotripsy (ESWL). The youngest patient observed with obstructive stones necessitating urologic intervention was 7 months old; the oldest was 63 years old and subsequently underwent bilateral ESWL.

### Kidney function

**Fig. 2:**
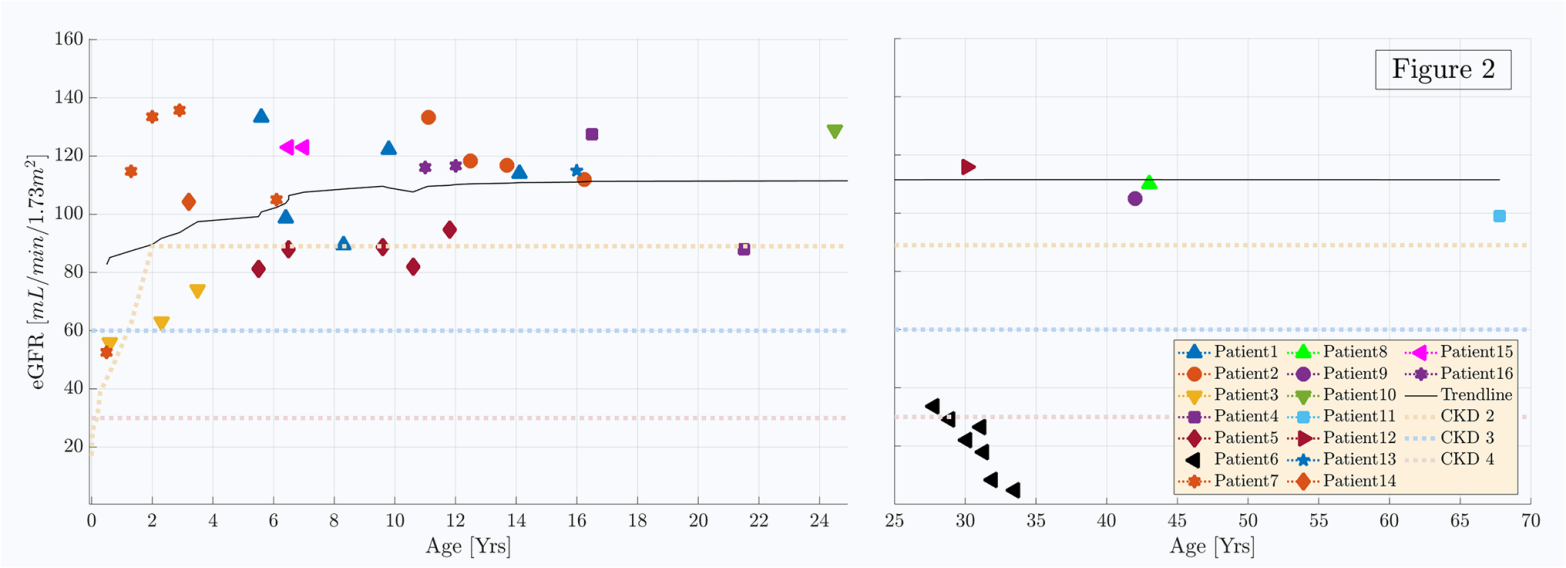
The changes in eGFR with age, in the reported cohort. The boundaries of CKD 2, 3, and 4 are illustrated by the dotted lines. Each individual is represented with a unique symbol. There are 2 children with a reduced GFR (CKD stages 2 and 3) during follow-up, and one adult who developed CKD stage 5

Kidney function was normal throughout follow-up in all patients except four (Fig. 2; Table 3): one patient who developed CKD stage 5 at the age of 28 years (patient 6) and three more patients (patients 3, 4, and 5). Patient 6 presented with recurrent kidney stones from the age of 7 months. At the age of 27 years, after several years with no follow-up, he presented with chronic kidney failure stage 4–5 (eGFR ~ 18 mL/min/1.73 m^2^) and massive proteinuria. Kidney biopsy showed focal mesangial sclerosis and focal chronic tubulo-interstitial changes with few oxalate deposits. Within a year of the kidney biopsy, the patient started peritoneal dialysis and subsequently underwent kidney transplantation at 34 years of age. Patient 3 had eGFR of 74 mL/min/1.73 m^2^ at 5 years of age (last follow-up) and had mild symptoms of kidney stone disease with no nephrocalcinosis and no obstructive stones necessitating urologic intervention. Her urinary oxalate-to-creatinine ratio was high in the one sample documented (0.7 mmol/mmol). Patient 4 had eGFR of 88 mL/min/1.73 m^2^ on his last visit. He had recurrent urolithiasis and underwent several urologic interventions until adulthood. Patient 5 had a fluctuating eGFR but this may have been due to concurrent stones throughout the follow-up period.

### Genetic analysis

Thirteen of the 16 patients were found to be homozygous for the c.944_946del, (p. Glu315del, *RCV000000046*) in *HOGA1* (NM_138413.3) causing PH3.

Two brothers were identified as homozygous for a distinct pathogenic variant, C.208C > T (p.Arg70*), *RCV000186476*, in the *HOG1A* gene. They live in a separate village (patients 13–14) and are part of a different community compared to the other patients in our cohort.

Patient 4 was found to be a compound heterozygote for biallelic PVs in *HOGA1* (NM_138413.4); the c.944_946del, (p. Glu315del, *RCV000000046*); and the c.119C > A **(**p.Pro40His, RCV001840929.2).

### Genetic screening

We screened 1167 healthy women from four villages between 2013 and 2022 and found 90 carriers, a carrier frequency of 1:13. We identified four genetically homozygous asymptomatic women (Table 1, patients 8, 9, 10, 12).

### Heterozygous patients

Three patients were found to be heterozygous for the c.944_946 del in *HOGA1* PV and had urinary symptoms. One patient had small asymptomatic kidney stones in infancy that resolved later. This patient presented with restlessness at the age of 4 months and had small bilateral hyperechoic kidney papillae (3 sites) that resolved at the age of 7 years. There was no hematuria. The patient was asymptomatic after the first presentation and remained asymptomatic throughout follow-up. The second patient presented at the age of 4 years with recurrent urinary tract infections, a dilated kidney pelvis, and a significantly smaller left kidney. On voiding cysto-ureterogram at the age of 7 years, she had low-grade reflux. Sonographic follow-up did not show any kidney stones or nephrocalcinosis. These two patients underwent whole genome sequencing which yielded no additional PV. The third patient (#4) suffered recurrent nephrolithiasis from infancy until adulthood and eventually was found to be compound heterozygote with two pathogenic variants in *HOGA1* on whole genome sequencing.

## Discussion

### Natural history of PH3

PH3 has been reported as a disease of childhood, with some recent studies indicating progression of stone disease into adulthood, and several cases describing CKD stage 5 [3–7].

The current study adds to the understanding of the progression and natural history of PH3, especially beyond childhood. The cohort includes 16 PH3 patients, 15 homozygous and one compound heterozygous, who were diagnosed at different ages (0–63 years) and followed up for a period of 0.5–20 years (median 6.25 years). Of the entire cohort, 4 patients were diagnosed at adulthood by screening (prenatal screening or after the diagnosis of a family member) and all of them were clinically asymptomatic. The remaining 12 patients (11 children and one adult) had kidney stones at presentation. The youngest patient had kidney stones at the age of 2 months and the oldest had a kidney stone event at the age of 63 years. Seven patients required urologic intervention for kidney stones (age range 7 months to 63 years). One patient progressed to CKD stage 5 and 2 more patients had CKD stage 2 at the last follow-up. The remaining 13 patients had normal kidney functions at the last visit. Our genetic screening project included 1167 healthy women at child-bearing age, from four genetically isolated villages, and 90 were found to be PH3 carriers, yielding a carrier frequency of 1:13 in this population.

Our findings indicate that PH3 can be variable in presentation and disease progression; stones may present up to the fifth decade of life and require intervention. In contrast, some patients may be diagnosed through prenatal tests or population screening, and, in our cohort, these individuals were found asymptomatic. This report also concurs with recent studies that reported possible deterioration in kidney function throughout life [3–7]. In three cases (18.8%), there was a significant decline in kidney function: two patients had CKD stage 2 at the last visit (patients 3 and 4) and one patient reached CKD stage 5 (patient 6). Our findings are like the report of the OxalEurope where 21.4% of the patients reached CKD stage 2 or higher [3].

Our cohort typically presented early in life (median age one year and age range of 0.16–2.5 years in pediatric patients) with symptoms of urolithiasis (e.g., hematuria, pain, and/or urinary tract infection). These findings are like the OxalEurope cohort where the median age of the first symptom was 1.9 years [3]. Most children were asymptomatic after the age of seven years, except for two patients who had recurrent stone events through adulthood, one (patient 4) at the age of 20 years and one (patient 11) at 63 years (Table 3).

### Kidney stones in our cohort

In our cohort, we found kidney stones and elevated urine oxalate levels through adulthood with possible kidney function deterioration. Of the entire cohort, six patients were adults at the last follow-up (ages 21.5–63 years old). Of these, four showed normal kidney function and normal kidney ultrasound at the last follow-up, but two adults (patients 4 and 11) reported kidney stones requiring urological intervention at the age of 20 and 63 years, respectively. Similar results were reported in the OxalEurope study of PH3, where 7 of 15 adult patients had recurrent kidney stones [3]. Moreover, we found two reports of PH3 patients who developed CKD stage 5, both in old age and after a long history of recurrent nephrolithiasis [4, 6].

These findings led the authors to suggest that although PH3 symptoms are more apparent in childhood years, a predisposition exists to a continuation of kidney stones at any age. This finding emphasizes the need for more intensive follow-up in the adult PH3 population.

### Crystal nephropathy in PH3

Patient 6, who presented at a young age with nephrolithiasis and was diagnosed as neither PH1 nor PH2 (a genetic diagnosis of PH3 was confirmed later), presented 25 years later with kidney failure (serum creatinine 3.78 mg%) and massive proteinuria. The proteinuria was glomerular with total protein excretion of 4063 mg in 24 h and urinary protein-to-creatinine ratio of 2.32 mg/mg, and albuminuria with albumin-to-creatinine ratio of 1.58 mg/mg and 24 h urinary albumin excretion of 2764 mg. Serum albumin was in the normal range. Immune serology tests (anti-nuclear antibodies, complement proteins C3, C4) were normal. Kidney ultrasound showed small-sized kidneys — the right kidney length was 7 cm and the left kidney length was 8.5 cm (the patient was 30 years old at that time).

Kidney biopsy showed 4 glomeruli: one with global sclerosis, one with mesangial sclerosis, and two normal glomeruli. Interstitial fibrosis and tubular atrophy (IFTA) of 25% with no vascular changes were noted. There was one suspected calcium oxalate deposit. On electron microscopy, there was 50% podocyte foot process effacement. The interpretation was focal mesangial sclerosis and chronic tubulointerstitial changes.

The patient started a trial of steroids, and the proteinuria declined from urinary protein-to-creatinine ratio of 1.58 mg/mg to 0.73 mg/mg with no significant change in serum creatinine. The patient had an infectious complication (septic arthritis of the hip) that resulted in rapid progression to kidney failure, and the steroids were stopped. The patient started peritoneal dialysis and later underwent kidney transplantation. At present, the kidney graft is functioning well, as reported by the patient who is now being followed in another medical center.

The histologic findings in patient 6 may be consistent with crystal nephropathy induced by oxalate deposits in the kidneys. In vitro experiments suggest that exposure of kidney cells to calcium oxalate crystals causes tubular damage and inflammation and scarring of the kidney parenchyma [15]. The process includes internalization of the crystals by the cells and adhesion of crystals to the cells. The kidney cells affected by calcium oxalate crystals showed changes in gene expression that induce fibrosis, inflammation and proliferation, and other changes in the extracellular matrix.

These in vitro findings were also observed in the kidney tissue of hyperoxaluric patients. The increased urinary oxalate excretion appears to cause recurrent clinical stone events in some patients, and progressive CKD caused by the oxalate-induced crystal nephropathy, i.e., interstitial inflammation and fibrosis that are caused by oxalate crystal deposition in the kidney parenchyma. This is like observations made in other hereditary crystal nephropathies such as those associated with PH1 and the high dihydroxyadenine excretion in APRT deficiency, Dent disease, enteric hyperoxaluria, and, to a lesser degree, cystinuria [15, 16]. Previous case reports have shown that a high enteric oxalate level may cause CKD stage 5 or acute kidney injury with the kidney biopsies showing glomerular sclerosis and interstitial fibrosis [17, 18]. Moreover, a patient with PH2 was reported to have partial glomerulosclerosis and focal sclerosis and recurrence of the disease upon the transplant [19].

Therefore, patient 6 provides important information. It is of significance to note that this patient underwent several urological interventions throughout childhood, including ESWL, that may have caused kidney scarring and predisposition to CKD. The patient was not followed between 4 and 29 years of age and returned to the nephrology consultant due to heavy proteinuria (glomerular) and kidney failure at the age of 29 years. The biopsy that was taken at the age of 29 years when the patient reached CKD stage 5, showed only one calcium oxalate crystal, less than expected in a case of crystal nephropathy. On the other hand, it might be the late outcome of crystal nephropathy caused in his childhood. Interstitial fibrosis was observed in 25% of the specimen, a lower proportion than we expected if the kidney damage was due to crystal nephropathy alone. Hence the diagnosis of crystal nephropathy as the main cause of CKD stage 5 in this patient is still in doubt, in the opinion of the authors. The evidence that supports crystal nephropathy in this patient is the clinical history of recurrent nephrolithiasis and urologic interventions due to PH3, but the histology is less convincing.

This case is very complicated and the etiology of CKD stage 5 is not clear. The patient might suffer from two different diseases when the PH3 caused crystal nephropathy with chronic changes and another immune-mediated disease caused the glomerular proteinuria and the deterioration of kidney function to kidney failure. It might still be explained by the kidney injury that the PH3 caused in his childhood and the small kidneys which led to FSGS (focal segmental glomerulosclerosis) in adulthood.

### “PH3-like phenotype” in heterozygous carriers

We identified one heterozygote patient for the PV who had nephrolithiasis in infancy that resolved after several years, like the typical PH3 phenotype. Urine oxalate was normal by semi-quantitative test (urinary organic acid profile which demonstrated no elevated oxalic acid) at the age of 5 months, and 24-h urine collection for oxalate was normal (0.27 mmol/24 h) at the age of 6 years. Urine calcium-to-creatinine ratio was normal in repeated samples and 24-h urinary calcium excretion was also within normal range. The urinary amino acid profile was normal (to exclude cystinuria). In 2015, Allard et al. described three heterozygous parents of PH3 offspring with symptoms of urolithiasis [5]. Our heterozygote patient underwent whole genome sequencing looking for a second genetic variant to exclude the possibility of compound heterozygosity or a deletion in *HOGA1* or other genetic cause for urolithiasis.

These data may indicate that *HOGA1* may not act as a completely recessive gene but rather might have some disease expression even within the heterozygous community. It is difficult to demonstrate a firm association between the stone disease and the heterozygosity in our cohort. To confirm this, further follow-up of more heterozygous patients is required.

### A genetic island of PH3

According to published genomic data, the worldwide prevalence of PH3 is 1:136,000, with PH3 carrier frequency at 1:185 [20, 21]. In a previous study, we found a significantly higher carrier rate of 1:13 among a genetically isolated population in the Golan Heights [22]. This finding led the Israeli National Health Department to endorse a genetic screening program for the PVs in this high-risk population [23]. Considering the rarity of PH3 worldwide, we have determined a genetic isolate for PH3 including undiagnosed presymptomatic patients and one of the largest groups studied to date. The high rate of carriers might serve as a platform for further studies related to clinical and biochemical markers in homozygotes and heterozygotes and their influence on possible kidney or other diseases in adulthood.

### The strength of our study

PH3 is a rare autosomal recessive kidney disease which is difficult to diagnose. Here we report on a genetic isolate of PH3, including 16 patients, 15 from the same ethnic background and a founder PV, and the same geographical and cultural environment. These ideal circumstances enable us to analyze the phenotypic spectrum of the patients and to follow the natural history of the disease alongside age with minimal “background noise.” Our cohort adds significantly to the existing information related to other cohorts described in the literature.

### The limitations of our study

There are several limitations to this study, the first being the small sample size of our adult cohort. Although the 15 new homozygous and one compound heterozygous patients reported here add significantly to the existing cohorts described in the literature, it is still not a large enough cohort to allow us to distinguish between subgroups in the cohort and make significant statistical deductions.

Most of our patients come from a similar genetic background and carry the same biallelic genetic variants, except for patients 4, 13, and 14 who live in a different geographical area/village. This may illustrate the phenotypic differences seen among patients with identical genetic variants due to possible modifier genes or environment but does not determine genotype/phenotype correlations with different *HOGA1* genetic variants.

Finally, there is missing data in our clinical follow-up description. As this was a retrospective study, the laboratory test results were collected from medical files, and the tests were not planned upfront as part of the study design. Most of the urine oxalate tests were done by urine collection in different laboratories and the creatinine values were not available in the test report; hence, we could not calculate creatinine excretion for body weight as a marker for urine collection validity. There was also missing laboratory data of urine metabolite levels (HOG, DHG, or 4OHGlu) because genetic testing is more readily available than metabolic profiling in our center. The diagnosis of PH3 was confirmed by genetic testing in all cases.

Nevertheless, since the follow-up goes back 20 years, missing data are unavoidable. Still, we could extract some important observations such as kidney function and kidney stone events in our PH3 patients until the last follow-up.

## Conclusions

PH3 is a less severe kidney stone disease than PH1 and most patients become asymptomatic by adulthood. Nevertheless, in some cases, symptoms continue through adulthood and may cause CKD, and may still have a high burden of kidney stones. Kidney stones in both childhood and adulthood should therefore always be evaluated for PH3. Pediatric and adult nephrologists should be aware and follow their PH3 patients clinically, biochemically, and imaging-wise through adulthood and provide nonspecific therapy for kidney stones even if the patients are asymptomatic.

A larger cohort of PH3 adult patients is needed to further develop a comprehensive understanding of the natural clinical progression of PH3 and determine evidence-based guidelines for follow-up and treatment.

Considering the high carrier rate found among the unique genetically community in the Golan Heights, we suggest that PH3 is underdiagnosed in this high-risk population. Although the cohort is composed of a homogenous population (with the same founder PV), we determined variable clinical presentations, suggesting that there might be a modifier gene or environmental factor that influences the phenotype.

Finally, patients found to be heterozygous for a genetic variant in *HOGA1* should be further evaluated for a second biallelic causative genetic variant which might confirm the diagnosis of PH3 and should be carefully followed up for clinical manifestations including kidney stones, flank pain, or even reduced eGFR.

## Supplementary Information

Below is the link to the electronic supplementary material.

Graphical abstract (PPTX 693 KB)

## Data Availability

The datasets generated during and/or analyzed during the current study are available from the corresponding author upon reasonable request. All genetic variants cited here are deposited and have a publicly available clinvar #.
