# Supplementary material for: Primary hyperoxaluria type 3: from infancy to adulthood in a genetically unique cohort
Source: Pediatr Nephrol. 2024 Oct 30;40(3):731–41. doi: 10.1007/s00467-024-06536-w (PMC11753311; doi:10.1007/s00467-024-06536-w)
Supplement: Supplementary file 1 — Graphical abstract (PPTX 693 KB) [file 467_2024_6536_MOESM1_ESM.pptx]

## Slide 1
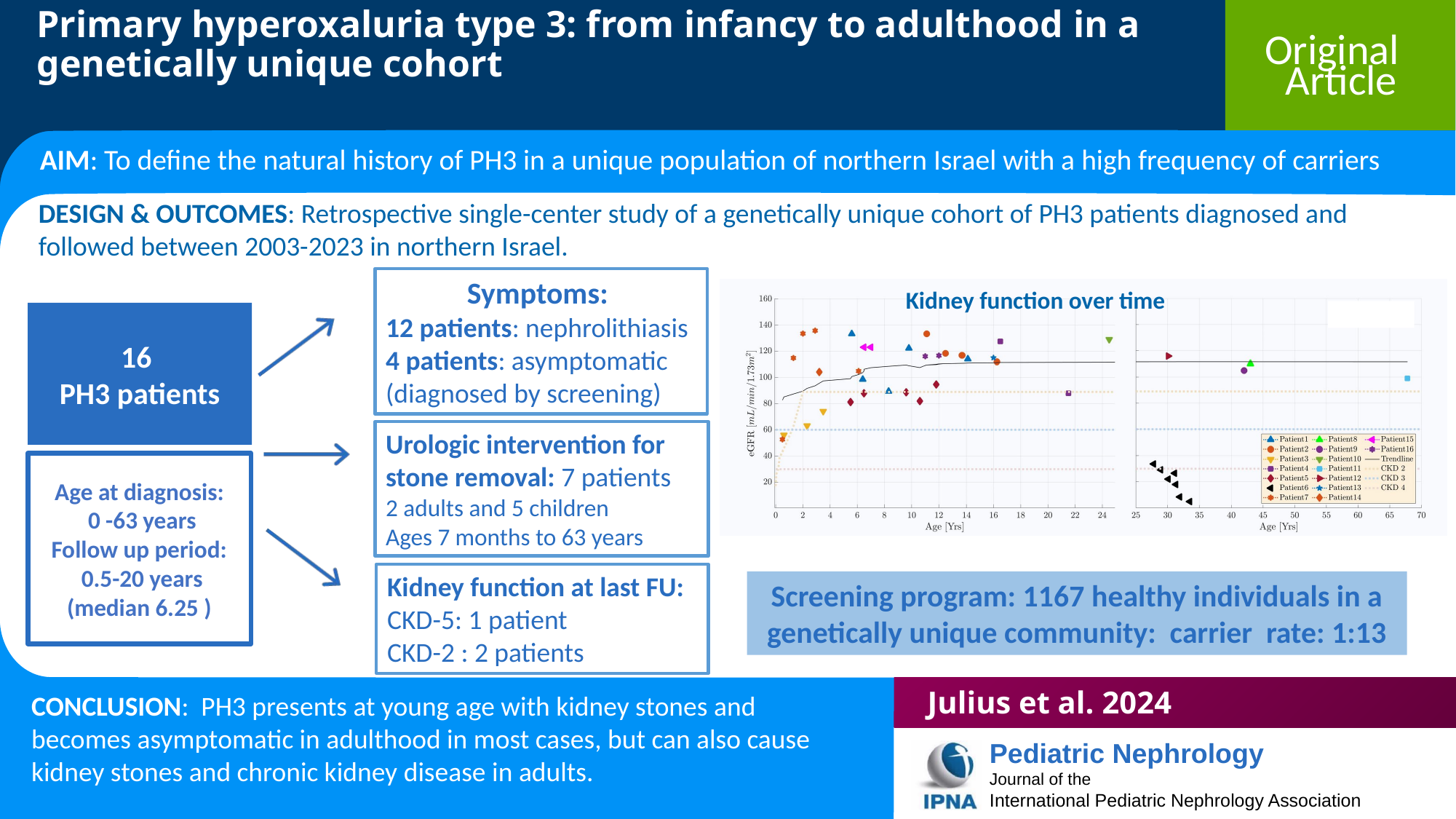

Primary hyperoxaluria type 3: from infancy to adulthood in a genetically unique cohort
AIM: To define the natural history of PH3 in a unique population of northern Israel with a high frequency of carriers
DESIGN & OUTCOMES: Retrospective single-center study of a genetically unique cohort of PH3 patients diagnosed and followed between 2003-2023 in northern Israel.
Symptoms:
12 patients: nephrolithiasis
4 patients: asymptomatic (diagnosed by screening)
Kidney function over time
16
PH3 patients
Urologic intervention for stone removal: 7 patients
2 adults and 5 children
Ages 7 months to 63 years
Age at diagnosis:
 0 -63 years
Follow up period:
 0.5-20 years (median 6.25 )
Kidney function at last FU:
CKD-5: 1 patient
CKD-2 : 2 patients
Screening program: 1167 healthy individuals in a genetically unique community: carrier rate: 1:13
Julius et al. 2024
CONCLUSION: PH3 presents at young age with kidney stones and becomes asymptomatic in adulthood in most cases, but can also cause kidney stones and chronic kidney disease in adults.
